# Pancreatic Head Mass from Metastatic Meningeal Hemangiopericytoma

**DOI:** 10.1155/2000/532304

**Published:** 2000-12-22

**Authors:** Bin S. Teh, Hsin H. Lu, Darshana N. Jhala, Imran Shahab, G. Rush Lynch

**Affiliations:** ^1^ Department of Radiation Oncology, Baylor College of Medicine, One Baylor Plaza, 165B, Houston, Texas 77030, USA; ^2^ Department of Pathology, Baylor College of Medicine, Houston, Texas, USA; ^3^ Department of Medical Oncology, Baylor College of Medicine, Houston, Texas, USA

## Abstract

*Patients or subjects*. A patient with long history of meningeal hemangiopericytoma was reported.

*Methods*. A case report on meningeal hemangiopericytoma with a literature review was presented.

*Results*. The patient has multiple local recurrence as well as distant metastases.This is the first case report of metastatic meningeal hemangiopericytoma causing compression of the pancreatic head.The patient also has biopsy-proven pulmonary metastases.The patient received both local and systemic therapy.

*Discussion*. It is important to recognize the distinctive features differentiating meningeal hemangiopericytoma from meningioma. The positive impact of clinico-pathological correlation on patient management is emphasized.


*Sarcoma* (2000) **4**, 169–172

ORIGINAL ARTICLE

### Pancreatic head mass from metastatic meningeal hemangiopericytoma

BIN S. TEH,[1] HSIN H. LU,[1] DARSHANA N. JHALA,[2] IMRAN SHAHAB[2] & G. RUSH LYNCH[3]

[1]*Department of Radiation Oncology,* [2]*Department of Pathology and* [3]*Department of Medical Oncology, Baylor College of Medicine, Houston, Texas, USA*

**Abstract**

*Purpose.* To illustrate the propensity of meningeal hemangiopericytoma to spread extraneurally, as a distinction to the ordinary meningioma.
*Patients or subjects.* A patient with long history of meningeal hemangiopericytoma was reported.
*Methods.* A case report on meningeal hemangiopericytoma with a literature review was presented.
*Results.* The patient has multiple local recurrence as well as distant metastases. This is the first case report of metastatic meningeal hemangiopericytoma causing compression of the pancreatic head. The patient also has biopsy-proven pulmonary metastases. The patient received both local and systemic therapy.
*Discussion.* It is important to recognize the distinctive features differentiating meningeal hemangiopericytoma from meningioma. The positive impact of clinico-pathological correlation on patient management is emphasized.

**Key words:** *meningeal hemangiopericytoma, pancreatic head metastases, radiotherapy*

### Introduction

Meningeal hemangiopericytoma (MH) is a rare tumor. The incidence of this malignant neoplasm ranges from 2 to 4% of that of meningiomas,[1,2] and makes up only less than 1% of all central nervous system (CNS) tumors.[2] It was postulated that MH arises from meningeal capillary pericytes or precursor cells with angioblastic tendencies.[3,4] The difficulty in recognizing MH as a separate entity from meningioma may have contributed to its low incidence. It is important to recognize this tumor because of its propensity to spread extraneurally, as a clear distinction to the ordinary meningioma, which has significant impact on both management and prognosis. Herein, we report a case of MH with multiple local recurrence as well as distant metastases. To our knowledge, this is the first report, in the English literature, of metastatic MH presented as a mass compressing the head of the pancreas.

### Case report

A 48-year-old African–American man was referred to the Department of Radiation Oncology for consideration of palliative radiotherapy to his brain. The consultation request form stated 'the patient has long history of meningioma, status post local resection times three. He also had pancreatic cancer with lung metastases, status post Whipple procedure. Currently, he has more headaches and imaging study showed a mass'. The referral service was concerned that the brain lesion might be from a pancreatic primary.

During the patient's first visit to the Department of Radiation Oncology, he stated that there was deterioration of his left-sided weakness and right-sided vision in addition to the headache. These were the same symptoms he initially presented 7 years ago. A head computed tomography (CT) scan was then performed that showed a mass in the right occipito-parietal area. The patient subsequently underwent craniotomy and resection. Intraoperatively, the tumor was noted to be very vascular and invading the sagittal sinus. Final pathology reported benign meningioma of angioblastic type. The patient was told that the tumor was benign. His symptoms improved post-operatively and he did not receive any adjuvant treatment such as radiotherapy. He remained symptom free until 2 years ago, when he presented to another hospital with headache. Neuro-imaging (both CT and magnetic resonance imaging (MRI)) showed a

Correspondence to: Hsin H. Lu, M.D., Baylor College of Medicine, One Baylor Plaza, 165B, Houston, Texas 77030. Tel: 713-790-2637; Fax: 713-793-1300; E-mail: sclark@bcm.tmc.edu

1357-714X print/1369-1643 online/00/040169-04 © 2000 Taylor & Francis Ltd
*DOI: 10.1080/13577140020025887*

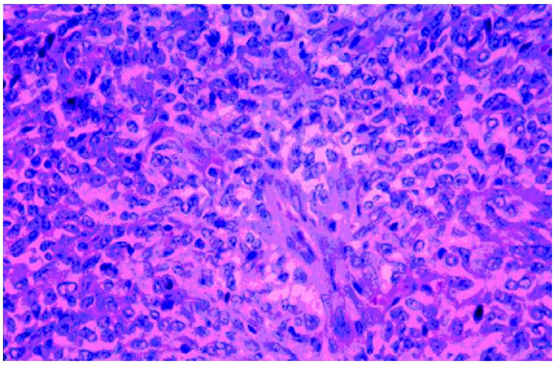

**Fig. 1.** *Meningeal hemangiopericytoma shown on high-power field.*

recurrent mass in the right occipito-parietal region that enhanced well with gadolinium, and there were multiple flow voids. An angiogram with vascular embolization was performed pre-operatively in an attempt to decrease vascularity. The patient underwent a second craniotomy and resection of the mass. A subgaleal drain was used because of the vascularity. Pathology reported meningeal hemangiopericytoma (Fig. 1). The postoperative course was complicated by removal of the retained fragment of the subgaleal drain, an emergent decompression of an intracerebral hemorrhage in the right surgical bed, resection of the residual tumor and repair of a subgaleal cerebro-spinal fluid leak. The patient recovered eventually without any neurological sequelae. Again, the patient did not receive any adjuvant treatment.

One year ago, the patient presented to the same hospital with abdominal pain and jaundice. Physical examination revealed an icteric conjunctiva and a soft non-distended abdomen with some degree of epigastric tenderness. There were no palpable masses, organomegaly or lymphadenopathy. Chest X-ray was normal. CT scan (Fig. 2) of the abdomen demonstrated a 3 cm mass compressing the head of the pancreas causing intra- and extra-hepatic biliary tract dilatation, suggestive of carcinoma. No other abnormality was noted. The patient then underwent exploratory laparotomy and Whipple procedure. Pathology initially reported undifferentiated malignant neoplasm. Further studies including immuno-

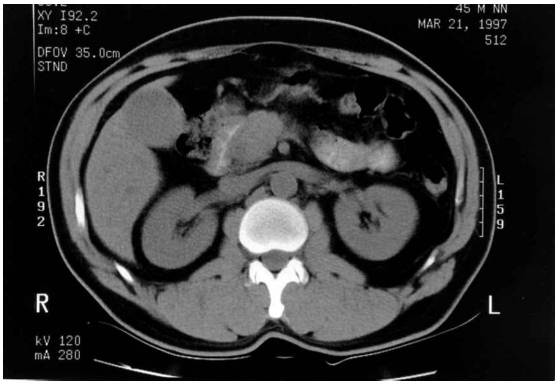

**Fig. 2.** *Computed tomography scan of the abdomen showing a 3 cm mass compressing the pancreatic head.*

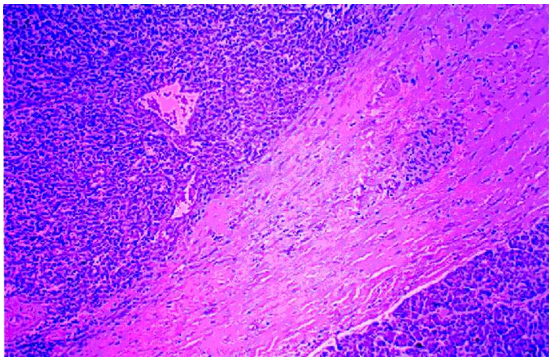

**Fig. 3.** *Low-power field shows meningeal hemangiopericytoma (left) separated from the normal pancreatic tissue (right) by connective tissues.*

histochemistry in our hospital confirmed that the pancreatic head mass was indeed hemangiopericytoma. The mass was purely extra-pancreatic in origin and the pancreatic tissue was normal (Fig. 3). The patient again recovered but was then lost to follow-up.

The patient has now returned with a 2-month history of intermittent headache, increasing left-sided weakness and worsening of his right vision. Physical examination revealed the patient was awake and oriented with decreased right visual acuity. Other cranial nerves were otherwise normal. He had unsteady gait with decreased power and sensation on his left-sided extremities. Other examinations were normal. Laboratory tests including liver function were within normal limits. MRI of the brain (Fig. 4) showed a large $5 \times 6 \times 5$ cm$^3$ inhomogeneous mass in the right occipito-parietal region. The mass was extra-axial with a dural tail and contained a large cystic component. It extended across the falx to the left and also invaded the superior sagittal sinus. Chest X-ray

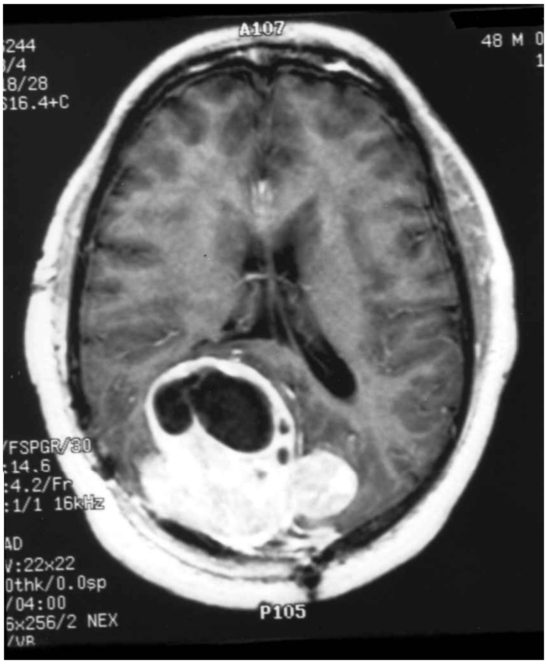

**Fig. 4.** *Axial image of a magnetic resonance imaging scan shows a large right occipito-parietal mass.*

and chest CT revealed multiple round densities in the lung parenchyma including the left lingula, right peri-hilar and right lung base. There was no significant lymphadenopathy. CT of the abdomen was normal without any metastatic disease. There was no bony metastasis on bone scan.

The patient was started on decadron with improvement of his symptoms. A CT-guided biopsy of the lung nodules was performed. The pathology showed metastatic hemangiopericytoma. The patient was to undergo further resection of the intra-cranial recurrent mass followed by adjuvant radiotherapy. Systemic chemotherapy comprising adriamycin and ifosfamide was also planned in view of its sarcoma-like behavior.

### Discussion

It is very unusual for primary CNS tumors to spread outside the craniospinal neural axis.[5] Extraneural metastasis is, however, common in hemangiopericytoma.[2] The other CNS tumor to metastasize in this manner is medulloblastoma.[6] The most common sites of metastases in descending frequency are bone, lung and liver.[2,7,8] To the best of our knowledge, this is the first case, in the English literature, of metastatic hemangiopericytoma presented as a mass compressing the head of the pancreas, mimicking carcinoma of pancreas. The patient actually had obstructive jaundice and underwent Whipple procedure. However, there is still a small probability that this intra-abdominal mass could be second primary hemangiopericytoma arising from the pericapillary cells in the local blood vessels rather than metastasis from the distant meningeal hemangiopericytoma. The other case of metastatic MH reported by Guthrie *et al.*[2] was in the retroperitoneum but no exact location was specified. It was even more interesting to note in the surgical specimen that all pancreatic tissues were normal and the intra-abdominal mass noted on the CT and ultrasonography (US) was purely extra-pancreatic in origin. The systemic dissemination of primary CNS tumors via ventriculo-peritoneal (VP) shunts, although uncommon, has been reported.[5,9,10] Other proposed mechanisms include neurosurgical intervention resulting in permeation of neoplastic cells to the vascular and lymphatic systems,[9] and treatment-related immunosuppression.[6] In this case, there was no placement of VP shunt and the patient was not immunosuppressed. However, he underwent four surgical interventions (for resection of recurrent tumor, removal of retained drain fragment, decompression of intracranial hemorrhage and repair of subgaleal cerebrospinal fluid leak, respectively) at the time of first local recurrence. This may have been the mechanism by which the malignant cells gain access into the lympho-vascular space to spread distantly.

This case further illustrates several distinctive features differentiating MH from the meningioma. MH is more common in males (55–70%) as compared with meningioma (35%), with the average age at diagnosis between 38 and 42 years.[1,2] The patient was 41 years old when he was first diagnosed. MH has an aggressive behavior and tends to recur both locally and distantly. The median recurrence-free interval was between 40 and 50 months, with an actuarial 5-, 10-, and 15-year recurrence rate of 65, 76 and 87%, respectively.[2,8] The first local recurrence occurred approximately 50 months after initial diagnosis in this patient. MH tends to re-recur at shorter intervals thereafter, as evidenced in this case. The patient recurred systemically 14 months later after first local recurrence, and further local and distant (lung) recurrences took place in another 17 months. It is important to know the natural history of MH and long-term follow-up is needed. The pathologic features of various specimens obtained at different times were similar. This is in contrast to that of ordinary meningioma, which usually shows malignant progression in cytological features at recurrence.

There is still no consensus on the terminology and histogenesis of MH. The nomenclature of angioblastic meningioma (hemangiopericytic type) was used in the first pathologic diagnosis and MH was used in the subsequent pathologic diagnoses. The malignant cell arises from either peri-capillary cells or pericytes of Zimmerman and can occur wherever capillaries are present. In the World Health Organization's classification of 1979, these tumors were included as a subgroup of meningioma, but for some authors they should not be classified as meningiomas. In contrast to the ordinary meningioma, MH tends to recur both locally and distantly. It was interesting to note that, in this case, the pathologist at the outside hospital reported the resected abdominal mass as undifferentiated malignant neoplasm. The pathologist most likely has no knowledge of the patient's previous history of MH. However, after review of the pathologic specimen here in our hospital, the diagnosis of MH is made. This again stressed the importance of clinico-pathological correlation that has direct impact on patient management. More recently, advances in cytogenetics[11] and immunohistochemistry[12] have provided additional tools to differentiate MH from the ordinary meningioma. Using cytogenetic studies, monosomy or partial deletion of chromosome 22 was demonstrated in 66% of ordinary meningioma but none in MH. The immunotyping studies of MH included positivity of vimentin (85%), factor XIIIa (78%) in individual scattered cells, Leu-7 (70%), and CD34 (33%) in a weak, patchy pattern. The ordinary meningioma characteristically expressed epithelial membrane antibody (80%) and S-100 protein (80%); CD34 reactivity (60%) was patchy and weak. Significant reactivity for P53 protein was detected in 5% of meningioma but in 52% of MH.[12]

Surgery is the gold standard for the initial management of MH.[13] If MH is suspected, arteriography should be performed with the possibility of embolization to reduce the risk of intra-operative hemorrhage. A gross total resection should at least be attempted at the time of initial surgery. Adjuvant radiotherapy is strongly recommended if resection is incomplete. In a recent Mayo Clinic series, surgery again was shown to be important for the successful treatment of patients with recurrent MH.[14] This series also found radiosurgery played a role in the treatment of smaller recurrent CNS lesions. Radiotherapy was helpful in the management of unresectable tumors and metastatic disease. Adriamycin-based chemotherapy was administered in seven patients (21%) and there was only one partial response that lasted 8 months. In view of his age and reasonable performance status, as well as long natural history of disease, the patient in this case was recommended to undergo further neurosurgical intervention, followed by radiotherapy to maximize the local control. He would also receive systemic chemotherapy comprising adriamycin and ifosfamide, frequently used in treating soft tissue sarcoma metastasizing to the lung.

Last but not least, this case illustrates the importance of good history taking and reviewing of all past medical records including previous pathological slides. The patient was referred to the radiotherapy department as having a stable meningioma and widespread metastatic carcinoma of the pancreas. He was not offered surgical intervention to the brain. However, after a thorough history taking and review of medical records, pathological slides including those from another hospital and additional immunohistochemistry studies, the patient's clinical picture became clear and he was managed appropriately.

### References

1 Chan RC, Thompson GB. Morbidity, mortality, and quality of life following surgery for intracranial meningiomas. *J Neurosurg* 1984; 60:52–60.

2 Guthrie BL, Ebersold MJ, Scheithauer BW, Shaw EG. Meningeal hemangiopericytoma: histopathological features, treatment, and long-term follow-up of 44 cases. *Neurosurgery* 1989; 25(4):514–22.

3 Stout AP, Murray MR. Hemangiopericytoma. *Ann Surg* 1942; 116(1):26–33.

4 Horten BC, Urich H, Rubinstein LJ, Montague SR. The angioblastic meningioma: a reappraisal of a nosological problem. *J Neurol Sci* 1977; 31:387–410.

5 Hoffman HJ, Duffner PK. Extraneural metastases of central nervous system tumor. *Cancer* 1985; 56:1778–82.

6 Rochkind S, Blatt I, Shadeh M, Goldhammer Y. Extracranial metastases of medulloblastoma in adults: literature review. *J Neurol Neurosurg Psychiatry* 1991; 54:80–6.

7 Jääskeläinen J, Servo A, Haltia M, Wahlström T, Valtonen S. Intracranial hemangiopericytoma: radiology, surgery, radiotherapy, and outcome in 21 patients. *Surg Neurol* 1985; 23:227–36.

8 Schroder R, Firsching R, Kochanek S. Hemangiopericytoma of the meninges. II. General and clinical data. *Zentralbl Neurochir* 1986; 47:191–9.

9 Berger MS, Baumeister B, Geyer JR, Milstein J, Kaney PM, LeRoux PD. The risks of metastases from shunting in children with primary central nervous system tumors. *J Neurosurg* 1991; 74:872–7.

10 Campbell AN, Chan HSL, Becker LE, Daneman A, Park TS, Hoffman HJ. Extracranial metastases in childhood primary intracranial tumors. *Cancer* 1984; 53:974–81.

11 Zattara-Cannono H, North MO, Gambarelli D, *et al.* The contribution of cytogenetics to the histogenesis of meningeal hemangiopericytoma. *J Neuro-Oncol* 1996; 29:137–42.

12 Perry A, Scheithaur BW, Nascimento AG. The immnophenotypic spectrum of meningeal hemangiopericytoma: a comparison with fibrous meningioma and solitary fibrous tumor of meninges. *Am J Surg Pathol* 1997; 21(11):1354–60.

13 Brunori A, Delitala A, Oddi G, Chiapetta F. Recent experience in the management of meningeal hemangiopericytomas. *Tumori* 1997; 83(5):856–61.

14 Galanis E, Buckner JC, Scheithaur BW, Kimmel DW, Schomberg PJ, Piepgras DG. Management of recurrent meningeal hemangiopericytoma. *Cancer* 1998; 82(10):1915–20.
